# Supplementary material for: Genetic and morphological diversity in populations of Annona senegalensis Pers. occurring in Western (Benin) and Southern (Mozambique) Africa
Source: PeerJ. 2023 Aug 8;11:e15767. doi: 10.7717/peerj.15767 (PMC10416773; doi:10.7717/peerj.15767)
Supplement: Supplemental Information 1 [file peerj-11-15767-s001.docx]

**Table S:** Bioclimatic variables

| Short name | Longname | Unit |
| --- | --- | --- |
| Chelsa_bio | mean annual air temperature | °C |
| Chelsa_b_1 | mean diurnal air temperature range | °C |
| Chelsa_b_2 | Isothermality | °C |
| Chelsa_b_3 | temperature seasonality | °C/100 |
| Chelsa_b_4 | mean daily maximum air temperature of the warmest month | °C |
| Chelsa_b_5 | mean daily minimum air temperature of the coldest month | °C |
| Chelsa_b_6 | annual range of air temperature | °C |
| Chelsa_b_7 | mean daily mean air temperatures of the wettest quarter | °C |
| Chelsa_b_8 | mean daily mean air temperatures of the driest quarter | °C |
| Chelsa_b_9 | mean daily mean air temperatures of the warmest quarter | °C |
| Chelsa_b_10 | mean daily mean air temperatures of the coldest quarter | °C |
| Chelsa_b_11 | annual precipitation amount | kg m^-2^ |
| Chelsa_b_12 | precipitation amount of the wettest month | kg m^-2^ |
| Chelsa_b_13 | precipitation amount of the driest month | kg m^-2^ |
| Chelsa_b_14 | precipitation seasonality | kg m^-2^ |
| Chelsa_b_15 | mean monthly precipitation amount of the wettest quarter | kg m^-2^ |
| Chelsa_b_16 | mean monthly precipitation amount of the driest quarter | kg m^-2^ |
| Chelsa_b_17 | mean monthly precipitation amount of the warmest quarter | kg m^-2^ |
| Chelsa_b_18 | mean monthly precipitation amount of the coldest quarter | kg m^-2^ |
